# DEAD-box helicase eIF4A2 inhibits CNOT7 deadenylation activity

**DOI:** 10.1093/nar/gkz509

**Published:** 2019-06-10

**Authors:** Hedda A Meijer, Tobias Schmidt, Sarah L Gillen, Claudia Langlais, Rebekah Jukes-Jones, Cornelia H de Moor, Kelvin Cain, Ania Wilczynska, Martin Bushell

**Affiliations:** 1 Medical Research Council (MRC), Toxicology Unit, University of Cambridge, Hodgkin Building, Leicester LE1 9HN, UK; 2 School of Pharmacy, University of Nottingham, University Park, Nottingham NG7 2RD, UK

## Abstract

The CCR4–NOT complex plays an important role in the translational repression and deadenylation of mRNAs. However, little is known about the specific roles of interacting factors. We demonstrate that the DEAD-box helicases eIF4A2 and DDX6 interact directly with the MA3 and MIF domains of CNOT1 and compete for binding. Furthermore, we now show that incorporation of eIF4A2 into the CCR4–NOT complex inhibits CNOT7 deadenylation activity in contrast to DDX6 which enhances CNOT7 activity. Polyadenylation tests (PAT) on endogenous mRNAs determined that eIF4A2 bound mRNAs have longer poly(A) tails than DDX6 bound mRNAs. Immunoprecipitation experiments show that eIF4A2 does not inhibit CNOT7 association with the CCR4–NOT complex but instead inhibits CNOT7 activity. We identified a CCR4–NOT interacting factor, TAB182, that modulates helicase recruitment into the CCR4–NOT complex, potentially affecting the outcome for the targeted mRNA. Together, these data show that the fate of an mRNA is dependent on the specific recruitment of either eIF4A2 or DDX6 to the CCR4–NOT complex which results in different pathways for translational repression and mRNA deadenylation.

## INTRODUCTION

The poly(A) tail at the 3′end of mRNAs plays a critical role in the life-cycle of an mRNA. Most mRNAs receive a poly(A) tail in the nucleus and regulation of the poly(A) tail length of each mRNA is subject to strict regulation (1). The poly(A) tail is bound by PABP, which acts both at the level of translation as well as mRNA stability via altering the poly(A) status of the mRNA (2–4). PABP also interacts with the eIF4F complex which in turn interacts with the cap structure at the 5′end of the mRNA resulting in mRNAs forming a closed loop conformation, stimulating translation efficiency. However, when an mRNA is targeted for deadenylation and decay, PABP can also interact with the CCR4–NOT complex which is critical for the removal of the poly(A) tail (5,6).

The CCR4–NOT complex plays an important role in many aspects of eukaryotic gene expression, but it is best known for its role in the translational repression and deadenylation of mRNAs (7). The CCR4–NOT complex is recruited to mRNAs in diverse ways, such as via miRNAs, RNA modification and/or RNA-BPs (8–12). CCR4–NOT recruitment results in translational repression, deadenylation and degradation of an mRNA (7). The CCR4–NOT complex is a large multiprotein complex with several proteins assembled around the scaffolding protein CNOT1. Amongst these proteins are the deadenylases CNOT7/8 which in turn bind CNOT6/6L (13). These deadenylases collaborate with each other and PABP to remove the poly(A) tail of an mRNA (5,6). Other important subunits are CNOT3, which plays a role in mRNA surveillance and mRNA export from the nucleus, and CNOT9 which interacts with TNRC6, one of the main effectors of the miRNA pathway (14).

Recently, two DEAD-box helicases, eIF4A2 (15; unpublished data Wilczynska *et al.*) and DDX6 (16–19) have been identified and shown to play a critical role in miRNA mediated translational repression via the CCR4–NOT complex. eIF4A2 is a paralogue of eIF4A1 and although the amino acid sequences of both proteins are highly similar they have opposing functions, resulting in contrasting effects of the helicases in cancer (20). eIF4A1 levels are increased in several cancers and predict poor prognosis, whilst high eIF4A2 levels are linked to better survival rates (20). eIF4A1 and eIF4A2 also have distinct functions in viral replication (21). eIF4A1 binds to the MIF and MA3 domains of eIF4G and is required for unwinding of the secondary structure in the 5′UTR of an mRNA while the initiation complex scans for the start codon (22,23). Therefore, eIF4A1 plays an important role in the translation efficiency of an mRNA. However, eIF4A2 interacts with the CCR4–NOT complex instead and is critical for translational repression and destabilization of the mRNAs it binds (15,24,25). DDX6 also interacts with the CCR4–NOT complex and has been shown to bind directly to CNOT1 (16–18).

So far there is no consensus regarding the exact function of the helicases interacting with the CCR4–NOT complex. Different research groups have detected either a role for eIF4A2 (15,26,27; unpublished data Wilczynska *et al.*) or DDX6 in miRNA mediated translational repression (16–19). The exact role of eIF4A2 remains under discussion with some research showing that eIF4A2 is required for miRNA mediated repression (15) whilst other research suggests that miRNA mediated repression results in the dissociation of eIF4A (26,27). However, other research has suggested that eIF4A2 is not required for miRNA mediated repression (16,17,19,28). The use of different model systems and technical approaches complicates a direct comparison of these studies. Endogenous eIF4A2 binds preferentially to CNOT1 (15,25). However, when eIF4A2 is overexpressed it has a tendency to interact with eIF4GI rather than CNOT1 (17,27,29; unpublished data Wilczynska *et al.*). When eIF4A2 is overexpressed at high levels it becomes difficult to differentiate between eIF4A1 and eIF4A2 which could result in inaccurate interpretation of the interactions when obtained with overexpression approaches (unpublished data Wilczynska *et al.*). With the data available so far we are currently not able to explain the differences observed by the different research groups.

Here, we show that both DEAD-box helicases eIF4A2 and DDX6 as well as deadenylase CNOT7 are required for translational repression via CNOT1. We have analysed the domains of the central part of CNOT1 and determined that the MA3 and MIF domains are essential for maximal helicase binding and function. Both helicases bind CNOT1 directly and compete for binding. We identified another CNOT1 binding protein, TAB182, which can affect helicase selection into the CCR4–NOT complex. This has immediate consequences for the fate of the mRNA as we demonstrated that eIF4A2 and DDX6 have distinct and opposing effects on the ability of CNOT7 to deadenylate the targeted mRNA. eIF4A2 interferes with the ability of CNOT7 to deadenylate an mRNA whilst DDX6 stimulates CNOT7 activity. Consequentially, analysis of mRNAs bound to eIF4A2 showed that these mRNAs had a much longer poly(A) tail than mRNAs bound to DDX6. The data presented here shows that the poly(A) tail length of mRNAs bound by the CCR4–NOT complex is determined by the recruitment of either eIF4A2 or DDX6 to the CCR4–NOT complex and the subsequent impact on deadenylation.

## MATERIALS AND METHODS

### Constructs

pRLSV40 (pRL) and pGL3 were obtained from Promega. pRLSV40 let-7 (eight let-7 repeats) has been described previously (30). pGL3 intron was generated by inserting a PCR product generated with pRLSV40 as template and primers Intron-F1/R1 into the HindIII and NcoI sites of pGL3. The tethering constructs (N)HA-CNOT1-MA3-MIF-DUF, (N)HA-CNOT1-MA3-MIF and (N)HA-CNOT1-MIF were generated by cloning PCR products (primers CNOT1-F1/F2/F3/R1/R2/R3 and template NHA-CNOT1C into the EcoRI and NotI sites of HA-CNOT1C and NHA-CNOT1C. The mutants 4G23 and CAF were generated using the same primers with templates NHA-CNOT1Cmut4G2+4G3 and NHA-CNOT1CmutCAF. The tethering controls (N)HA-GFP were generated by cloning PCR products (primers GFP-F1/R1) into the EcoRI and NotI sites of HA-CNOT1-MIF and NHA-CNOT1-MIF. The tethering reporter Rluc-BoxB-HsL-HhR, the plasmids used as templates and vectors for the cloning were a gift from Witek Filipowicz (31,32). The coding sequence of *CNOT7* was amplified using primers CNOT7-F/R and cloned into pET-45b. PCRs were performed using KOD polymerase (Merck) according to the manufacturer's instructions. cDNAs corresponding to *eIF4A1* (primers TS3/TS4), *eIF4A2* (primers TS5/TS6), *DDX6* (primers TS7/TS8), *eIF4G-*MIF-MA3 (primers TS9/TS10), CNOT1-MIF (primers TS21/TS22) and *CNOT1*-MA3-MIF (primers TS19/TS22) were cloned into pET-SUMO vector using the BsaI and NotI restriction sites*. eIF4A2*^DAAD^ has been generated by site-directed mutagenesis using primers TS38/TS39. These PCRs were performed using Phusion polymerase (Thermo Fisher Scientific) according to manufacturer's instructions. For primer sequences see Supplementary Table S1.

### Cell lines, siRNAs and transfections and reporter assays

**Figure 1.**
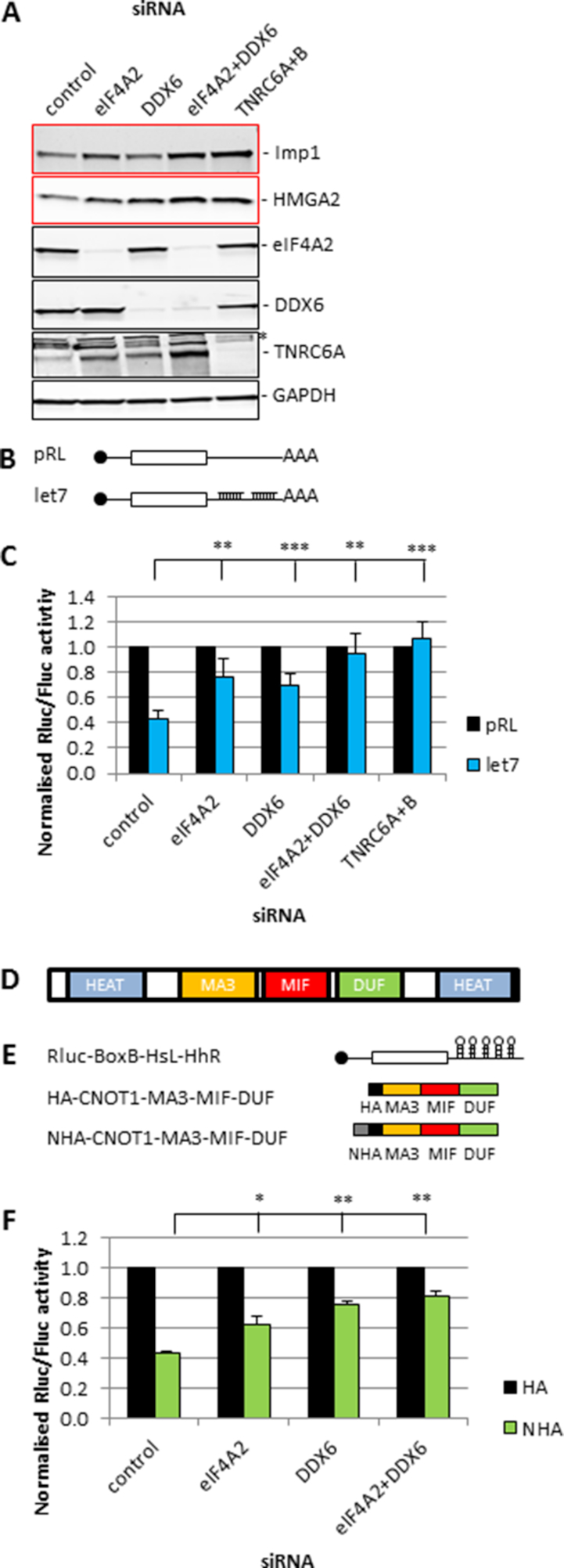
eIF4A2 and DDX6 are both required for CNOT1 mediated translational repression. (**A**) HeLa cells were transfected with siRNAs as indicated. Cells were lysed in RIPA buffer after 72 h. Lysates were analysed using western blotting. Representative blots are shown. GAPDH is a loading control. *Indicates a nonspecific band. (**B**) Schematic representation of constructs used in the let-7 reporter assay. (**C**) HEK293 cells were transfected with siRNAs and after 24 h with the plasmids from (B) as indicated. Cells were lysed in PLB after a further 24 h and analysed by luciferase assay. Luciferase activity was plotted as average ± sd, *n* = 4 biological repeats. Significance was calculated using a Student's *t*-test (paired, two-tailed); **P< 0.01, ***P< 0.001. (**D**) Schematic representation of the CNOT1 domains. (**E**) Schematic representation of the constructs used in the tethering assay. N-tag was required for tethering, HA tag was used for western blotting. (**F**) HeLa cells were transfected with siRNAs and with the plasmids from (E) after 24 h as indicated. Cells were lysed in PLB after a further 48 h and analysed by luciferase assay. Luciferase activity was plotted as average ± sd, *n* = 3 biological repeats. Significance was calculated using a Student's *t*-test (paired, two-tailed); *P< 0.05, **P< 0.01.

**Figure 2.**
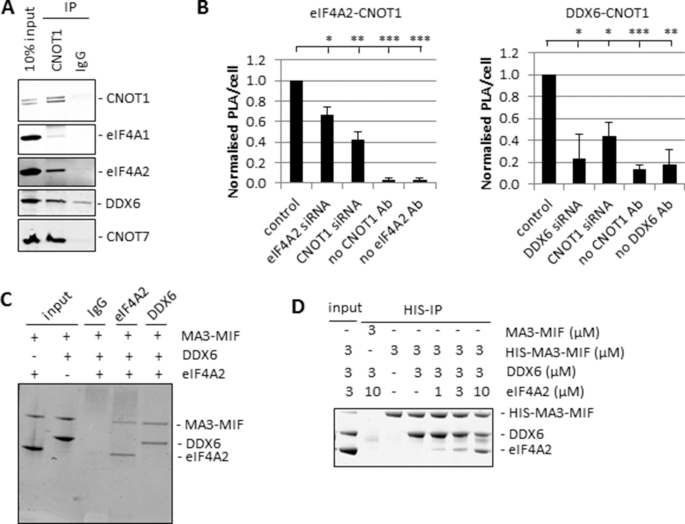
eIF4A2 and DDX6 both interact directly with CNOT1 and compete for binding. (**A**) Immunoprecipitation from untransfected HeLa lysates with CNOT1 antibody or IgG as indicated. Protein complexes were eluted from the beads using a CNOT1 peptide and analysed by western blotting. Representative blots are shown. (**B**) HeLa cells were transfected with siRNA and analysed by proximity ligation assay (PLA) as in Supplementary Figure S3A–B. PLA dots were counted using ImageJ and plotted as average number of PLA dots per cell ± sd, *n* = 3 biological repeats. Significance was calculated using a Student's *t*-test (paired, two-tailed); *P< 0.05, **P< 0.01, ***P< 0.001. (**C**) Recombinant eIF4A2 and DDX6 proteins were incubated with recombinant CNOT1-MA3-MIF and subsequently pulled down with the indicated antibodies and analysed by denaturing SDS-PAGE and Coomassie-staining. A representative gel is shown. (**D**) Recombinant proteins were mixed at increasing concentrations of eIF4A2 and assembled complexes were pulled down via the HIS-tag of CNOT1. Co-precipitated proteins were analysed by SDS-PAGE and Coomassie-staining. A representative gel is shown.

**Figure 3.**
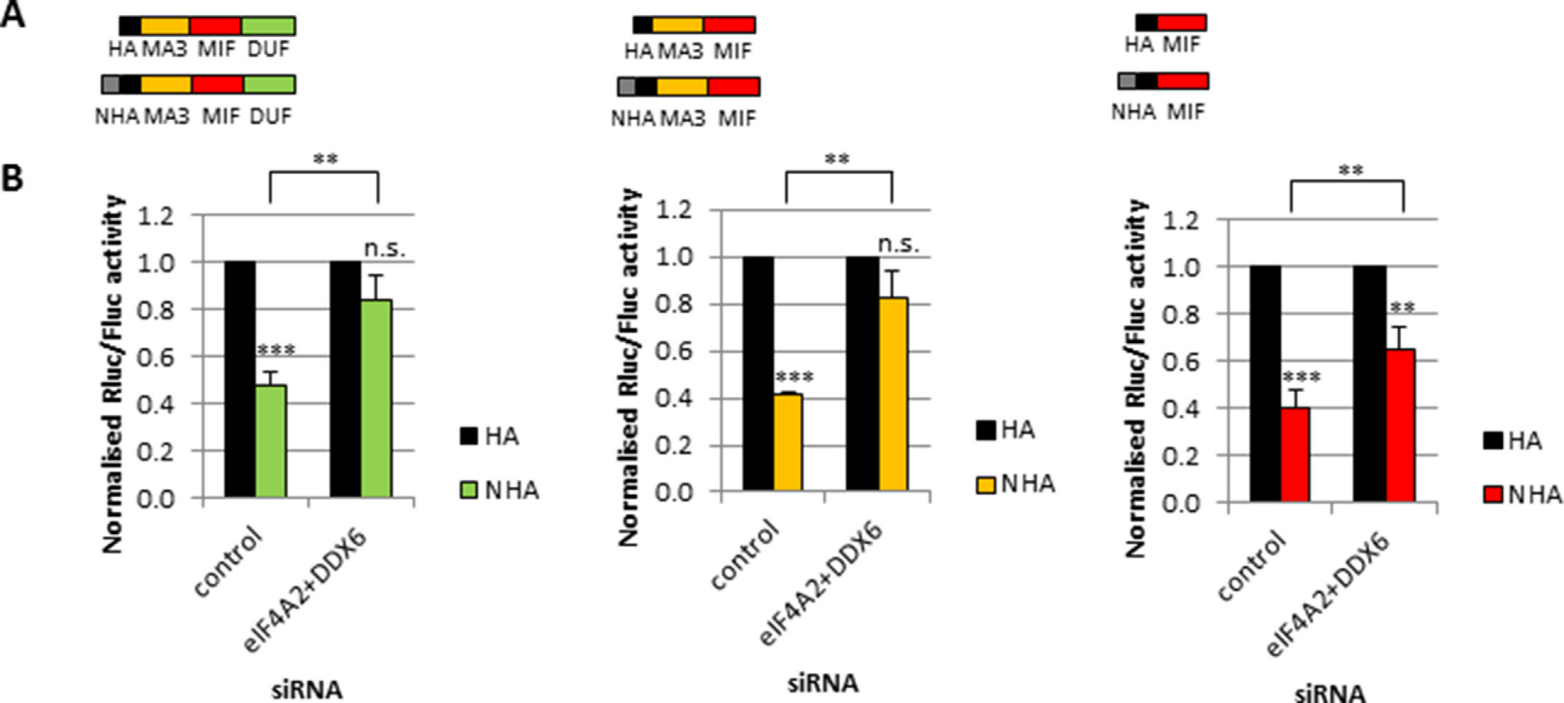
The MIF domain of CNOT1 is sufficient for maximum repression but both the MA3 and MIF domains are required for maximum helicase response and maximum helicase binding. (**A**) Schematic representation of constructs used in the tethering assay. (**B**) HeLa cells were transfected and analysed as in Figure 1F with the constructs depicted in (A) and analysed by luciferase assay, *n* = 4 biological repeats. Significance was calculated using a Student's *t*-test (paired, two-tailed); **P< 0.01, ***P< 0.001, n.s. = not significant.

**Figure 4.**
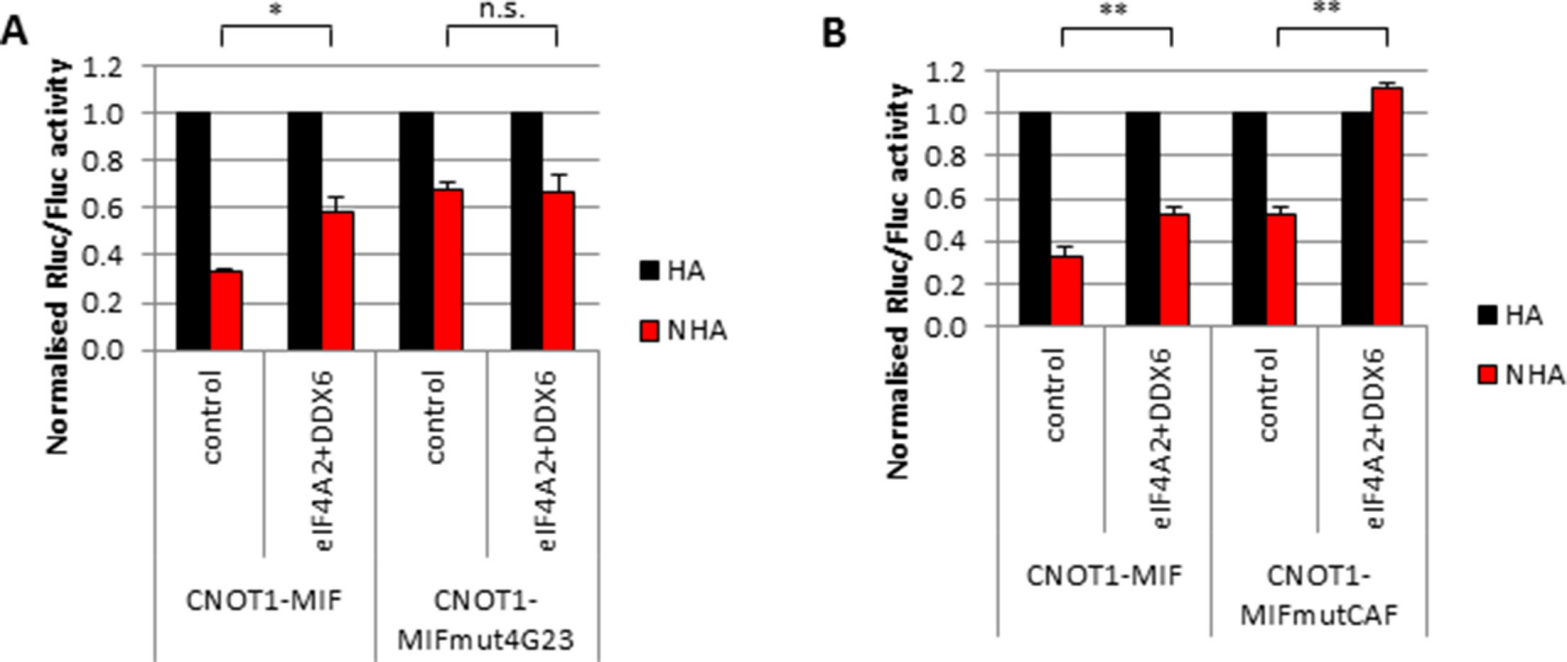
eIF4A2, DDX6 and CNOT7 are required for maximum repression via CNOT1. (**A**) HeLa cells were transfected and analysed as in Figure 1F with the constructs depicted in Figure 3A (right hand panel) and analysed by luciferase assay, *n* = 3 biological repeats. The CNOT1-MIFmut4G23 construct contains mutations that prevent helicase binding. (**B**) HeLa cells were transfected and analysed as in Figure 1F with the constructs depicted in Figure 3A (right hand panel) and analysed by luciferase assay, *n* = 3 biological repeats. The CNOT1-MIFmutCAF construct contains mutations that prevent CNOT7 binding. Significance was calculated using a Student's *t*-test (paired, two-tailed); *P< 0.05, **P< 0.01, n.s. = not significant.

**Figure 5.**
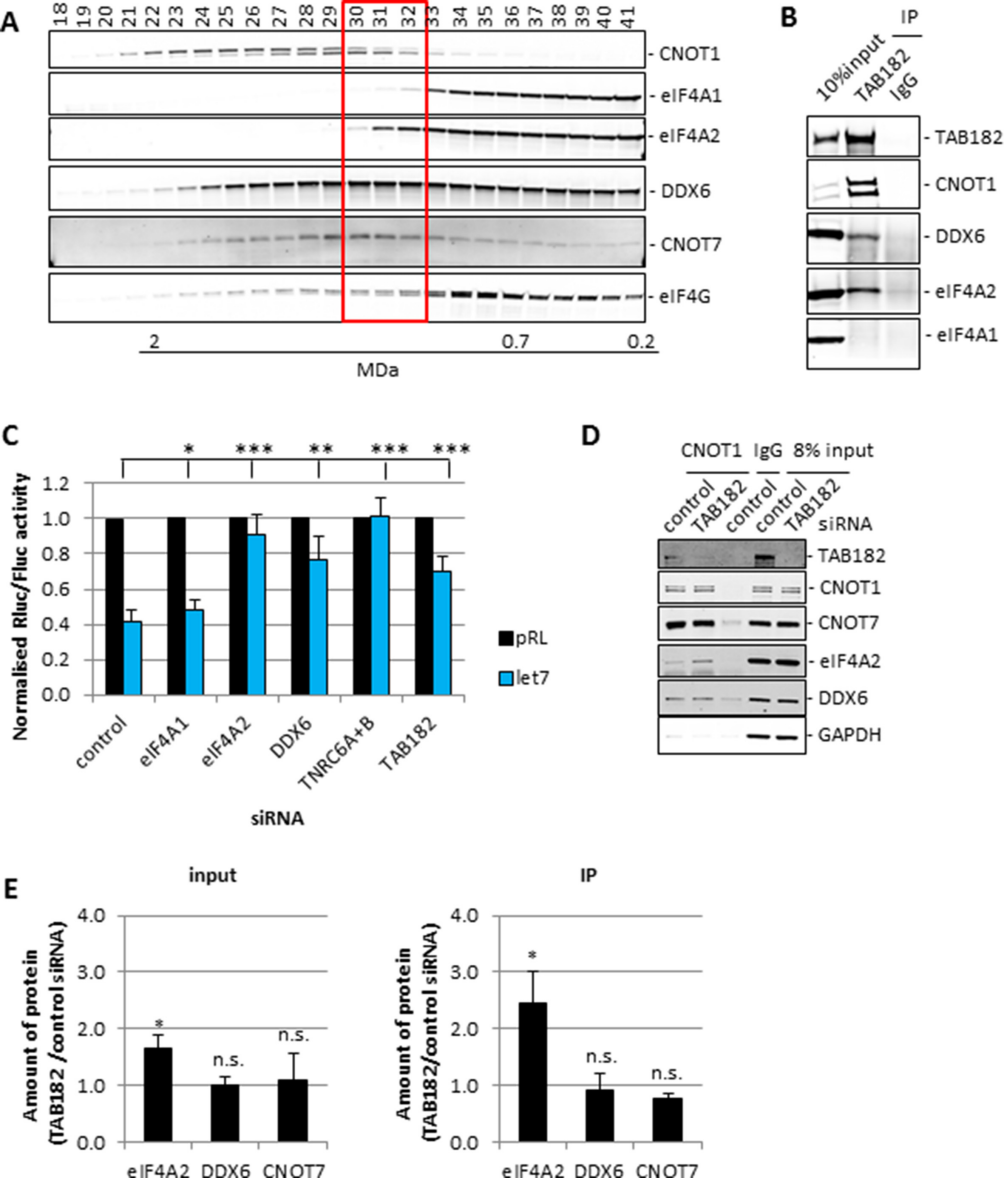
TAB182 affects eIF4A2 incorporation into the CCR4–NOT complex. (**A**) Gel filtration fractions 18–41 (from Supplementary Figure S7A) were analysed by western blotting to allow for detailed analysis of migration patterns. Representative blots are shown. (**B**) Immunoprecipitation from HeLa lysates with TAB182 antibody or IgG in the presence of RNaseA. Immunoprecipitates were analysed using western blotting. Representative blots are shown. (**C**) HEK293 cells were transfected and analysed as in Figure 1C, *n* = 4 biological repeats. Significance was calculated using a Student's *t*-test (paired, two-tailed); *P< 0.05, **P< 0.01, ***P< 0.001. (**D**) HeLa cells were transfected with siRNAs as indicated. Cells were lysed after 72 h and immunoprecipitated with the indicated antibodies. Protein complexes were eluted with CNOT1 peptide and analysed by western blotting. Representative blots are shown. GAPDH is a loading control. (**E**) Quantification of three biological repeats of the experiment shown in Figure 5D. Significance was calculated using a Student's *t*-test (paired, two-tailed); *P< 0.05, n.s. = not significant.

Two cell lines were used in this paper: HeLa and HEK293. Both were cultured in DMEM supplemented with 10% FBS and 2 mM l-glutamine. siRNAs (see Supplementary Table S2) were transfected on day 1 using 1 μl/ml (HeLa) or 2 μl/ml (HEK293 and Figure 5D, Supplementary Figure S7G) DharmafectI (Dharmacon). siRNA concentration varied between experiments: 30 nM (Figure 5C, Supplementary Figure S9), 2 × 30 nM (Figure 1A, C, Supplementary Figures S2A–B, S2E–G), 60 nM (Figures 2B, 4 and 8C, Supplementary Figures S3, S5 and S6), 2 × 60 nM (Figures 1F, 3, Supplementary Figure S2C–D), 120 nM (Figure 5D, Supplementary Figure S7G). Cells were transfected with plasmids on day 2 using 1μl/ml (, Supplementary Figure S5D: 2 ul/ml) Genejammer (Agilent). For let-7 reporter assays 40 ng/ml pRL/pRL-let-7 and 160 ng/ml pGL3-intron were used. For tethering assays 300 ng/ml (N)HA overexpression plasmid, 50 ng/ml BoxB reporter and 200 ng/ml pGL3-intron were used. For the tethering of (N)HA-GFP 0.3 ng/ml was used to obtain similar expression levels to (N)HA-CNOT1. pGL3-intron was used as a transfection control for all reporter assays. For overexpression of HA tagged proteins 1 μg/ml HA plasmid was used. Cells were harvested on day 3 (let-7 reporter, overexpression immunoprecipitations) or day 4 (endogenous targets, tethering) and lysed in 1× PLB, Trizol, RIPA or IP buffer. PLB and Trizol samples were immediately frozen at −80°C. Luciferase assays were performed as described previously (15). Protein concentrations were determined using Biorad Protein Assay.

### RNA, RT and qPCR

RNA from transfected cells, cleared lysate or gel filtration fractions was isolated using 1 ml of Trizol (Invitrogen) according to the manufacturer's protocol. RNA yield and purity (A260/280) was determined using a nanodrop 2000 (Thermo Scientific). Reverse transcription of 500 ng of total RNA was performed using random hexamers and SuperscriptIII (Invitrogen) following the manufacturer's instructions. qPCR was performed on a 7500 Fast Real Time PCR System (Applied Biosystems) using Fast Sybr Green Mastermix (Applied Biosystems) according to the manufacturer's protocol and analysed using 7500 software v2.3 (Applied Biosystems). Cycling conditions were as follows: denaturation for 20 s at 95°C followed by 40 cycles of 3 s at 95°C and 30 s at 60°C. For each biological repeat, three technical repeats were performed. For primers used see Supplementary Table S1. Renilla and Firefly plasmids used in the transfections contained an intron and primers were intron spanning. No RT and no template controls were included. The Renilla amplicon length was 106 bp (let-7 reporters) or 109 bp (tethering reporters) and the Firefly amplicon length was 107 bp. Renilla mRNA levels were normalized for Firefly mRNA levels. Meltcurves were determined for each experiment. Standard curves were obtained for each primer pair. Coefficients of determination were 0.9865–0.9996. PCR efficiencies were 97–108%. Tresholds were automatically determined by the 7500 software based on the signal obtained during cycles 3–15. Observed Ct values for experimental samples were on average 12 Ct values lower than no template controls.

### Gel filtration columns

Cytoplasmic HeLa lysates (Ipracell) were cleared by centrifugation for 15 min at 13 000 rpm at 4°C and fractionated by size-exclusion chromatography using a HiPrep 16/60 Sephacryl S-500 HR column connected to an AKTApurifier protein purification system (GE Healthcare Life Sciences). The column was eluted at 4°C with 5% (w/v) sucrose, 0.1% (w/v) CHAPS, 20 mM HEPES–NaOH, 5 mM DTT and 150 mM NaCl, pH 7.0, at 0.15 ml/min and 2 ml fractions collected.

### Recombinant protein purification

eIF4A1, eIF4A2, DDX6, eIF4G-MIF-MA3, CNOT1-MA3-MIF and CNOT1-MIF were heterologously produced in *Escherichia coli* BL21 (DE3) CodonPlus-RP as N-terminal 6xHis-SUMO-fusion proteins. CNOT7 was produced as N-terminal 6xHIS-tagged protein. Biomass was produced applying standard protocols for IPTG-induction. Cells were harvested, resuspended and lysed in buffer A (20 mM Tris–HCl, pH 7.5, 1 M NaCl, 30 mM imidazole, 10% (v/v) glycerol) supplemented with 1 mM PMSF and complete EDTA-free protease inhibitor cocktail (Roche). After centrifugation at 75 000 g supernatant was filtered (5 μm) and applied to HisTrap (GE Healthcare) affinity chromatography. Bound protein was eluted with a linear imidazole gradient. Pooled fractions were diluted in buffer B (20 mM Tris–HCl, pH 7.5, 10% (v/v) glycerol, 0.1 mM EDTA) and except from CNOT7 pools incubated with SUMO-protease for 1 h at 8°C for cleavage of the SUMO-tag. CNOT7 proteins were incubated with TEV instead. The protein solutions were further diluted with buffer B and eIF4A1, eIF4A2, eIF4A2^DAAD^ and CNOT1-MA3-MIF were applied to a ResourceQ (GE Healthcare) anion exchange and DDX6 and eIF4G-MIF-MA3 samples to Heparin affinity chromatography. Bound protein was eluted with a linear KCl gradient. Pooled fractions were further purified by size exclusion chromatography using a Superdex 200 column equilibrated in storage buffer (20 mM HEPES–KOH, pH 7.5, 100 mM KCl, 0.1 mM EDTA, 10% (v/v) glycerol, 1 mM Tris(2-carboxyethyl)phosphine). In case of eIF4G-MIF-MA3 and CNOT1-MA3-MIF the storage buffer contained 250 mM or 200 mM KCl respectively. Pooled fractions were concentrated, snap-frozen in liquid nitrogen and stored at −80°C. Protein concentrations were calculated from the absorbance at 280 nm (*A*_280_) using extinction coefficients 34 630 M^−1^ cm^−1^ (eIF4A1), 40 130 M^−1^ cm^−1^ (eIF4A2), 30 745 M^−1^ cm^−1^ (DDX6), 69 495 M^−1^ cm^−1^ (eIF4G-MIF-MA3), 23 950 M^−1^ cm^−1^ (CNOT1-MIF), 41 830 M^−1^ cm^−1^ (CNOT1-MA3-MIF) and 39 225 M^−1^ cm^−1^ (CNOT7) obtained from ExPASy server. All protein preparations showed an *A*_280_/*A*_260_ ratio of ≥1.9 indicating negligible amounts of contaminations by nucleic acids and nucleotides.

### Immunoprecipitations with recombinant proteins

To study CNOT1 interactions 3 μM CNOT1-MA3-MIF or CNOT1-MIF was incubated with 3 μM of eIF4A1, eIF4A2 and DDX6 for 1 h at room temperature in NP-buffer (20 mM HEPES/KOH, pH 7.5, 100 mM KCl, 1 mM MgCl_2_, 2 mM DTT, 0.1% (v/v) NP40). In CNOT1 competition experiments, reactions were performed in the presence of 5 μM ssRNA (AG)_10_ (IBA life science) and 1 mM AMPPNP (Sigma). For eIF4G interactions, 3 μM eIF4G was incubated with 3 μM of eIF4A1, eIF4A2 and DDX6 for 1 h at room temperature in NP-buffer. Per reaction 50 μl Dynabeads Protein G (Thermo Fisher Scientific) were washed twice with NP-buffer and coated with 5 μg of antibody (see Supplementary Table S3) for 10 min in NP-buffer at room temperature. Coated beads were washed 3× in NP-buffer and incubated with protein samples for 15 min at room temperature. Beads were washed 3× with NP-buffer, mixed with SDS gel loading buffer, heated at 95°C for 5 min and applied to denaturing polyacrylamide gel electrophoresis. After electrophoresis, gels were stained with Coomassie and bands visualized using a Licor Odyssey scanner.

### Immunoprecipitations with cell lysates and gel filtration fractions

HeLa or HEK293 cells were scraped in cold PBS and pelleted by centrifugation for 5 min at 4000 rpm at 4°C. Pellets were resuspended in IP buffer (5% (w/v) sucrose, 0.1% (w/v) CHAPS, 20 mM HEPES, 50 mM NaCl, pH 7.0) supplemented with 1× complete EDTA-free protease inhibitor cocktail (Roche) and 0.1% (v/v) ß-mercaptoethanol. Lysates where treated with 50 U/ml benzonase in the presence of 10 mM MgCl_2_ by rotation for 1 h at 4°C (Figure 5D only) or sonicated for 5 min followed by 10 min on ice. Lysates were cleared by centrifugation for 15 min at 4°C at 13 000 rpm and supernatants used in subsequent steps. When commercial cytoplasmic HeLa lysate (Ipracell) or gel filtration fractions were used the IP buffer was supplemented with 5 mM DTT instead and benzonase treatment/sonication was omitted. If the cells were treated with siRNA 200 μg protein was used per IP, for non-treated cells equal volumes of lysate were used. For immunoprecipitations on gel filtration fractions 0.4 ml of fractions 30–32 were used and total volumes were increased to 2.6 ml per sample. Per IP 25 μl Dynabeads protein G beads (Thermo Fisher Scientific) were incubated with 500 μl lysate in IP buffer for 1 h rotating at 4°C. Another 25 μl beads were blocked by incubating in 1% BSA and 0.1 mg/ml tRNA in IP buffer for 2 h rotating at 4°C. The pre-cleared lysates were transferred to new tubes and rotated in the presence of 10 μg antibody (see Supplementary Table S3) for 1 h at 4°C. A volume equivalent to 25 μl blocked beads was added to the lysate/antibody mix and rotated for a further 2 h. The beads were washed with 600 μl IP buffer for 3× 10 min and resuspended in SDS sample buffer. For some CNOT1 immunoprecipitations the protein complexes were eluted from the beads in 12 μg CNOT1 peptide (ProteinTech) in 60 μl 0.6× IP buffer for 30 min at 1600 rpm at 4°C. The eluate was then mixed with SDS sample buffer. For the immunoprecipitation of HA-tagged overexpressed proteins the same protocol was used with the substitution of HA beads (Pierce) for the Dynabeads and the protein complex was eluted using 120 μg HA-peptide (Thermo Fisher Scientific) in 60 μl 0.8× IP buffer for 10 min at 1600 rpm at 37°C.

### RNA immunoprecipitations

Per sample 8 × 10^6^ HEK293 cells were lysed in lysis buffer (20 mM Tris pH 7.5, 200 mM NaCl, 5 mM MgCl_2_, 0.5% (v/v) Triton-X100, 1× complete EDTA-free protease inhibitor cocktail (Roche), 1% BSA, 0.5 mM DTT, 5 mM NaF, 40 U/ml Riboblock (Thermo Fisher Scientific)). Lysates were cleared by centrifugation for 10 min at 4°C at 5000 rpm and supernatants used in subsequent steps. Six microgram of antibody (see Supplementary Table S3) were bound to 18 μl of Dynabeads protein G beads (Thermo Fisher Scientific) by rotating at 4°C for 2.5 h and subsequently the beads were washed three times with lysis buffer. Lysates were added and rotated for 30 min at 4°C. Beads were washed three times with lysis buffer and RNA was extracted with Trizol reagent (Invitrogen) followed by acid phenol extraction and ethanol precipitation.

### Proximity Ligation Assay (PLA)

siRNA treated HeLa cells were fixed for 10 min in 4% paraformaldehyde at 4°C. PLA was performed using the Duolink In Situ reagents (Sigma) according to the manufacturer's protocol with additional washes in PBS after fixing/blocking and additional washes in wash buffer A after primary antibody incubations. Blocking was done for 45 min at 37°C in blocking solution with 0.3% (v/v) Triton. Primary antibody incubations were performed for 1 h at room temperature. For antibody dilutions see Supplementary Table S3. The images were acquired on a Zeiss LSM 510 META inverted confocal microscope equipped with a multiphoton MaiTai, 488, 514, 543 and 633 nm laser lines using a 63×/1.4 oil Plan-Apochromat objective. At least 100 cells were counted per condition per experiment. ImageJ was used to count the number of PLA dots per cell.

### Mass spectrometry

LC–MS/MS was performed as described previously (33). Results were analysed in Scaffold (protein threshold 95%, minimum two peptides). Proteins with spectra counts in IgG or less than four spectra in either IP were removed. For the CNOT1 immunoprecipitation experiments, all peptides that were allocated to eIF4A1 also completely match the eIF4A2 sequence and therefore could have been derived from either protein. Although the MS did not enable us to differentiate between eIF4A1 and eIF4A2 due to the high similarity of these paralogues, since we used gel filtration fractions 30–32 which contain very little eIF4A1 these peptides have been allocated to eIF4A2.

### Electrophoretic mobility shift RNA-binding

25 nM Dy680- labelled (CAA)_6_CA RNA (IBA life science) was incubated with indicated proteins in binding buffer (20 mM HEPES/KOH, pH 7.5, 100 KCl, 1 mM MgCl_2_, 1 mM AMP-PNP, 1 mM TCEP, 0.1% DMSO) in 10 μl reactions for 60 min at 25°C. A final concentration of 2% (w/v) Ficoll-400 was added to the samples and complexes separated on 7% acrylamide-TB gels. Gels were scanned with Odyssey (Licor).

### Deadenylation assay

Recombinant proteins were incubated at room temperature at 3 μM in assay buffer (20 mM HEPES–KOH, pH 7.5, 100 mM KCl, 1 mM MgCl_2_, 2 mM DTT) in the presence of 1 mM AMPPNP (Sigma) and 1 μM fluorphor-labelled deadenylation substrate ssRNA Dy780-(CAA)_6_CA-C_20_-A_20_ (IBA life science). For competition experiments, 3 μM DDX6, 3 μM CNOT1 and indicated concentrations of eIF4A2 were preincubated with RNA and AMPPNP in assay buffer. After 1 h pre-incubation an aliquot was taken and mixed 1:1 with stop solution (0.5× TBE, 10 mM EDTA, 0.2% (w/v) SDS, 85% (v/v) formamide) and Dy680-(CAA)_6_CA-C_20_ marker ssRNA. Reactions were then started by the addition of the CNOT7 nuclease at a final concentration of 0.5 μM. At indicated time points aliquots were taken and mixed 1:1 with stop solution. Samples were heated for 2 min at 95°C and loaded on acrylamide–8M–urea TBE gels. After electrophoresis gels were incubated for 5 min in 10% (v/v) acetic acid and bands visualized using a Licor Odyssey scanner. Signals were quantified using Image Studio software (Licor) and the fraction of fully deadenylated product was plotted versus time using Prism GraphPad.

### Polyadenylation test (PAT)

Total RNA was deadenylated by incubating 10 μg RNA in RNase H buffer (75 mM KCl, 50 mM Tris–HCl pH 8.3, 3 mM MgCl_2_, 10 mM DTT) supplemented with 8 μg oligo(dT) and 10U of RNaseH (NEB) in a total volume of 100 μl for 1 h at 37°C. Deadenylated RNA was purified by phenol extraction and ethanol precipitation. RNA used for the PAT was deadenylated RNA, total RNA or RNA derived from RNA immunoprecipitation. The PAT anchor oligo was ligated onto the RNA by adding 200 ng RNA to the ligation mix (50 mM Tris–HCl pH 7.5, 10 mM MgCl_2_, 1 mM DTT, 10% (w/v) PEG8000, 1 μM PAT anchor oligo and 100 U of T4 RNA ligase 2—truncated KG (NEB) in a total volume of 10 μl) and incubated at 16°C overnight. Subsequently, the RNA was reverse transcribed in First Strand Buffer (50 mM Tris–HCl pH 8.3, 75 mM KCl, 3 mM MgCl_2_) supplemented with 5 mM DTT, 0.2 mM dNTPs, 1 μM PAT-R1 oligo and 200 U SuperscriptIII (Invitrogen) in a total volume of 50 μl. First the oligo and dNTPs were added and the RNA was denatured for 5 min at 65°C and cooled on ice. Then the remaining components were added and the mixture incubated for 1 h at 55°C, followed by 15 min at 70°C. For the RNA IP samples qPCR analysis was performed to determine relative amounts of the targeted mRNAs (for primers, see Supplementary Table S1). PCR products were then generated by two rounds of PCR. cDNA was amplified using GoTaq (Roche) according to the manufacturer's protocol, using 1 μl of cDNA (the RNA IP samples were diluted using the PCR results as guidance), PAT-R0 and gene-specific primer-PAT1 (see Supplementary Table S1) in the first round. One microliter of the resulting product was then amplified in a second round using PAT-R1 and gene-specific primer-PAT2 (see Supplementary Table S1). PCR program for both rounds: 5 min 95°C (hotstart), 40 cycles consisting of 1 min at 95°C, 1 min at 58°C and 2 min at 72°C, followed by 10 min at 72°C. The resulting PCR products were analysed on 4% high resolution agarose in TBE buffer (Sigma) containing SybrSafe (1:10 000) and stained post run with SybrGold (1:10 000 in TBE) if required. Gels were imaged using a GeneFlash Imager (Syngene).

### Determination of antibody affinity and protein concentration in lysate

To compare the affinities of the antibodies to their targets, specific amounts of the appropriate recombinant protein were separated on SDS-PAGE, transferred to the same membrane and detected using their specific antibodies and identical solution of secondary antibody. Band intensities were quantified with Licor software (ImageStudio) and plotted versus the protein amounts. The data were fit to a straight line and the slopes of the fits were compared between the individual antibodies to reveal their relative affinity. To determine the relative protein amount of eIF4A1 and eIF4A2 in HeLa lysate, different amounts of HeLa lysate were analysed the same way side-by-side with a reference titration of recombinant protein. The slopes of the fits were compared between reference protein and Hela lysate to determine the amount of eIF4A1 or eIF4A2 in the lysate.

### Statistical analysis

For reporter assays, PLA and quantification of protein levels three or four biological repeats (as indicated in the Figure legend for each experiment) were analysed using a Student's *t*-test (paired, two-tailed). Significance is indicated as follows: *P < 0.05, **P < 0.01, ***P < 0.001, n.s. = not significant.

## RESULTS AND DISCUSSION

### DEAD-box helicases eIF4A2 and DDX6 are both required for translational repression via the CCR4–NOT complex

To determine the importance of eIF4A2 and DDX6 for CCR4–NOT mediated translational repression we used three different strategies in two different cell lines (Figure 1, Supplementary Figures S1 and S2). Firstly, we knocked down both helicases individually or combined and showed the impact on protein levels of endogenous miRNA targets. Protein levels of Imp1 and HMGA2 were increased to varying degrees dependent on the helicase/target combination but were always elevated when both helicases were knocked down (Figure 1A). Secondly, we knocked down the helicases in combination with transfection of a luciferase reporter construct with or without let-7 target sites in the 3′UTR (Figure 1B). The presence of the miRNA target sites clearly repressed luciferase activity and this was partially reversed by knockdown of the individual helicases. Translation was completely restored after knockdown of both helicases together or knockdown of the positive control TNRC6A/B, which is required for the recruitment of the CCR4–NOT complex by miRNAs (Figure 1C; 34). Thirdly, we combined the helicase knockdowns with a tethering reporter. Tethering of the central region of CNOT1 (MA3-MIF-DUF; Figure 1D) has been shown to be sufficient for translational repression (Supplementary Figure S1; 16). We used a stabilized reporter which contains histone and ribozyme hairpins in the 3′UTR for stabilization of the reporter mRNA substituting for a poly(A) tail (Figure 1E). The reporter also contains BoxB hairpins in the 3′UTR for tethering of CNOT1. To study the effect of eIF4A2 and DDX6 on this reporter, we knocked down the helicases followed by tethering of the central region of CNOT1 to the stabilized reporter (Figure 1F). Again, both helicases were required for translational repression. Knockdowns were efficient and changes of mRNA levels were not responsible for the observed effects on protein levels (Supplementary Figure S2A–D). To exclude the possibility that these effects are siRNA specific we used alternative siRNAs for eIF4A2 and DDX6 and repeated the let-7 reporter experiment (Supplementary Figure S2E-G). The results were consistent, therefore the impact of the helicase knockdowns was not specific to the particular siRNAs used for eIF4A2 or DDX6 knockdowns. Taken together, these experiments showed that although individual contributions of eIF4A2 and DDX6 are context dependent, both are critical for translational repression via the CCR4–NOT complex.

### eIF4A2 and DDX6 interact directly with CNOT1 and compete for binding

Although it has been shown that both eIF4A2 (15,24) and DDX6 (16–18) can interact with the CCR4–NOT complex, how eIF4A2 interacts with the CCR4–NOT complex is unclear. Previous research has demonstrated the ability of the CNOT1 MIF domain to interact with the helicase DDX6 and the deadenylase CNOT7, albeit on opposing sides of the domain (16–18,35). The CNOT1 MIF domain has a similar structure to the MIF domain of eIF4G which is involved in eIF4A1 binding. To investigate the interaction between the CCR4–NOT complex and the DEAD-box helicases eIF4A1, eIF4A2 and DDX6 we performed endogenous CNOT1 immunoprecipitation experiments (Figure 2A). Both DDX6 and eIF4A2 interacted with CNOT1, but for eIF4A1 only a slight interaction was detected. The eIF4A1 and eIF4A2 antibodies have almost identical affinities for the proteins they are detecting (eIF4A1/eIF4A2 = 1.0; Supplementary Figure S3A). These results are in agreement with previously published data (15,25). We then employed proximity ligation assays (PLA) and confirmed that both eIF4A2 and DDX6 were in very close proximity of CNOT1 in the cell, suggesting a direct interaction (Figure 2B, Supplementary Figure S3B-D). Knockdown of one of the interacting proteins or omitting one of the primary antibodies significantly reduced the number of PLA dots (Figure 2B). The direct interaction was confirmed by immunoprecipitations using recombinant proteins (Figure 2C) which showed that both eIF4A2 and DDX6 interact directly with the central region of CNOT1. For this experiment we used the MA3 and MIF domains of CNOT1 which proved to be sufficient for binding to both helicases. To investigate in more detail the ability of the helicases to bind CNOT1, we performed *in vitro* competition assays and showed that eIF4A2 and DDX6 compete directly for binding to CNOT1 (Figure 2D). Previous research has only detected the interaction between CNOT1 and DDX6 (16,17), potentially because of the use of overexpression constructs rather than endogenous proteins in immunoprecipitation experiments.

To further define which domains of CNOT1 are reliant on the helicases for function we tethered different CNOT1 domains to the BoxB reporter construct, focusing on the region around the MIF domain (Figure 3A). The results showed that although the MIF domain alone is sufficient for maximum repression, both the MA3 and MIF domains were required for maximum response to the helicase knockdown, suggesting that both domains were required for optimal binding (Figure 3B, Supplementary Figure S4A-B). We used immunoprecipitations using recombinant protein to compare the interactions of the CNOT1 domains with eIF4A2 and DDX6. Both helicases bind with similar efficiency to CNOT1-MA3-MIF (Figure 2C). However, when repeating the same experiment with only the MIF domain we could not detect an interaction with either helicase (Supplementary Figure S4C). Previous research has observed an interaction between CNOT1-MIF and DDX6 (16–18), but this interaction was too weak to be detected under the conditions we used. However, these experiments clearly show that the MA3 and MIF domains were required for maximum binding to both eIF4A2 and DDX6 (Figure 2C, Supplementary Figure S4C).

### eIF4A2, DDX6 and CNOT7 are required and sufficient for translational repression

To confirm the requirement of both helicases for translational repression, we inhibited the interaction of the helicases with CNOT1 in two different ways: we compared the effect of the knockdown of the helicases on the tethering of the wildtype CNOT1-MIF domain with a CNOT1-MIF mutant domain that cannot interact with the helicases (CNOT1-MIFmut4G23; Supplementary Figure S5A; 16). The helicase binding mutant was not responsive to helicase depletion (Figure 4A, Supplementary Figure S5A–C), confirming that this mutant does not interact with the helicases and that this interaction is required for the helicases to function. To investigate why the MIF domain is resisting full derepression when eIF4A2 and DDX6 were knocked down we analysed a CNOT1 mutant that cannot bind the deadenylase CNOT7 (CNOT1-MIFmutCAF; Supplementary Figure S5D; 16). When combining the helicase knockdown with the CNOT7 binding mutant full translational activity was restored, demonstrating that eIF4A2, DDX6 and CNOT7 are required and sufficient for translational repression via the CNOT1-MIF domain (Figure 4B, Supplementary Figure S5D–F). To establish the individual contributions of each helicase to translational repression via the MIF domain, we knocked down the helicases followed by tethering of CNOT1-MIF (Supplementary Figure S6). The derepression after the individual knockdown of either helicase was limited, but knock down of both helicases together clearly increased translation levels of the tethering reporter. Combined, these results demonstrated that eIF4A2, DDX6 and CNOT7 together play a critical role in translational repression via the CCR4–NOT complex.

### TAB182 affects helicase incorporation into the CCR4–NOT complex

To further investigate CCR4–NOT complex composition we separated protein complexes from cytoplasmic HeLa lysate on a Sephacryl S-500 gel filtration column and analysed the resulting fractions by western blotting (Figure 5A, Supplementary Figure S7A and B). These showed clearly that the DEAD-box helicases eIF4A1, eIF4A2 and DDX6 have distinct migration patterns demonstrating that the helicases reside in separate multi-protein complexes. A portion of eIF4A2 and DDX6 proteins co-migrated with CNOT1 (fractions 30–32) whilst hardly any eIF4A1 was present in the same fractions as CNOT1 (Figure 5A). Quantification of eIF4A1 and eIF4A2 protein levels in fractions 30–32 as well as in unfractionated HeLa lysate allowed us to calculate the ratio of the eIF4A paralogues in the fractions. HeLa lysate contains 0.7 ng eIF4A1 and 0.04 ng eIF4A2 per μg lysate (Supplementary Figure S7C). In gel filtration fractions 30–32 the enrichment of eIF4A2 is 136× higher than eIF4A1 (Supplementary Figure S7D).Together these results show that fractions 30–32 contain approximately eight times more eIF4A2 than eIF4A1. Co-migration of CNOT1 and eIF4A2 is compatible but not proof that these proteins are part of the same complex in these fractions. Therefore, we confirmed the interaction of CNOT1 and eIF4A2 by CNOT1 immunoprecipitation from fractions 30–32 in the presence of RNaseA (Supplementary Figure S7E and F) showing that CNOT1 and eIF4A2 were indeed forming a complex. Performing the immunoprecipitation on lysate of cells treated with CNOT1 siRNA resulted in the loss of the complex showing the specificity of the interactions (Supplementary Figure S7G).

To identify any other proteins that are part of the eIF4A2–CNOT1 complex we separated protein complexes from cytoplasmic HeLa lysate on a Sephacryl S-500 gel filtration column followed by immunoprecipitations for CNOT1 and eIF4A2 from fractions 30–32 (Supplementary Figure S7H). The resulting samples were analysed by MS which resulted in 107 potential CNOT1–eIF4A2 complex binding proteins that were detected in both CNOT1 and eIF4A2 immunoprecipitations but did not show any background binding to IgG (Supplementary Table S4). To narrow down the number of candidates, the CNOT1 IP was repeated but complexes were eluted using the peptide used to raise the CNOT1 antibody (Supplementary Figure S7I). This resulted in a much longer list of CNOT1 binding proteins (Supplementary Table S5) as expected since CNOT1 is part of several large multi-protein complexes. However, combining the results of both experiments and selecting for any proteins that were detected in all immunoprecipitations but not in either of the IgG controls resulted in a list of 50 potential proteins that were in a complex with eIF4A2–CNOT1 (Table 1). Many of these proteins have previously been identified as RNA binding proteins (36). Some have also been identified as part of the CCR4–NOT complex such as CNOT3, COPA, PABP1, RFC4 and TAB182 (13). It is interesting to note that Lau *et al.* (13) also detected an interaction between several of the CNOT subunits and eIF4A. Although they identified this as eIF4A1, this may have been misannotated due to the extremely high similarity between eIF4A1 and eIF4A2, which makes it very difficult to distinguish between them by MS analysis.

**Table 1.** Consolidated MS data. MS data from Supplementary Tables S4 and S5 were ranked on total spectra/molecular weight with the highest number ranked 1 and combined

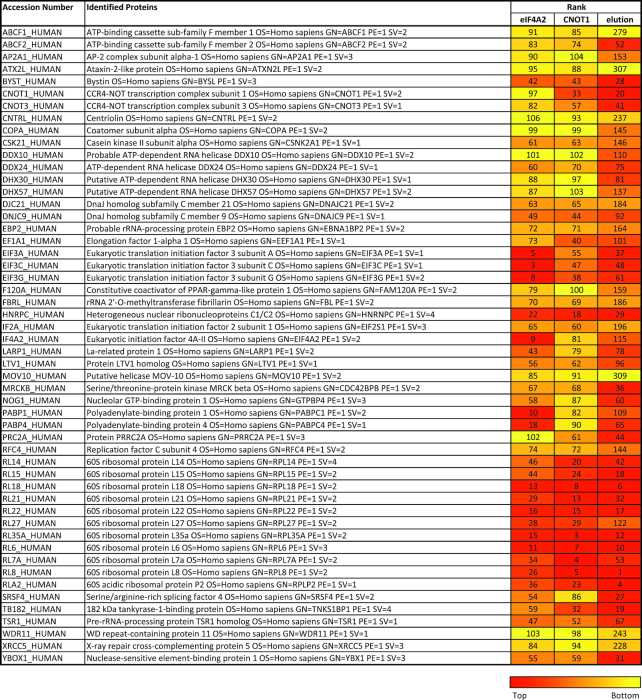

The interaction of several of the MS candidates was confirmed by immunoprecipitation followed by western blotting. All potential candidates tested were confirmed to interact with both CNOT1 and eIF4A2 (Supplementary Figure S8A) and to be present in fractions 30–32 after gel filtration (Supplementary Figure S8B) with the exception of the ribosomal proteins, RPL7a and RPL15, which showed background binding to IgG (Supplementary Figure S8A). The ribosomal proteins have therefore been excluded from further analysis. To identify any proteins that were interacting specifically with eIF4A2 we performed immunoprecipitations for eIF4A1 and eIF4A2 using HeLa lysate (Supplementary Figure S9A). Both antibodies precipitated comparable levels of eIF4A1 and eIF4A2 respectively as shown by the pan eIF4A antibody. This antibody has a similar affinity for eIF4A1 and eIF4A2, detecting eIF4A1 slightly more efficiently (eIF4A1/eIF4A2 = 1.5; Supplementary Figure S9B). This established that many of the proteins were interacting with both eIF4A1 and eIF4A2, as expected based on the high similarity of eIF4A1 and eIF4A2 amino acid sequence. TAB182 was the only new protein identified in the mass spectrometry experiments to precipitate with DDX6 and eIF4A2 but not eIF4A1 (Figure 5B, Supplementary Figure S9A), suggesting a role in miRNA mediated translational repression. TAB182 (also known as tankyrase 1 binding protein, TNKS1BP1) plays a role in DNA double strand break repair (37) and is involved in actin cytoskeleton rearrangement and cancer cell invasion (38). It has also been identified as part of the CCR4–NOT complex but its function therein remains unknown (13).

To confirm the role of TAB182 as part of the CCR4–NOT complex we knocked down TAB182 followed by transfection of the let-7 reporter constructs. The knockdown facilitated derepression in the let-7 reporter assay (Figure 5C, Supplementary Figure S9C and D). These results confirmed the involvement of TAB182 in miRNA mediated repression of translation. To investigate whether TAB182 had any effect on CCR4–NOT complex composition we immunoprecipitated CNOT1 following TAB182 knockdown. The interaction between CNOT1-CNOT7 and CNOT1–DDX6 remained the same (Figure 5D and E). Surprisingly, TAB182 knockdown increased eIF4A2 incorporation into the CCR4–NOT complex (Figure 5D and E). These results show that TAB182 has the ability to change CCR4–NOT complex composition, determining which helicase is associated with the CCR4–NOT complex.

### eIF4A2 inhibits CNOT7 deadenylation activity

To date, our knowledge regarding the role of the DEAD-box helicases in the CCR4–NOT complex is rather limited. In embryonic stem cells CNOT1 bound DDX6 is important for translational repression rather than mRNA destabilization (39) even though DDX6 bound mRNAs have slightly shorter poly(A) tails compared to total mRNA of HEK293 cells (40). Translational repression can be achieved via interaction of DDX6 with 4E-T which interacts with the 5′end of an mRNA (41–43).

**Figure 6.**
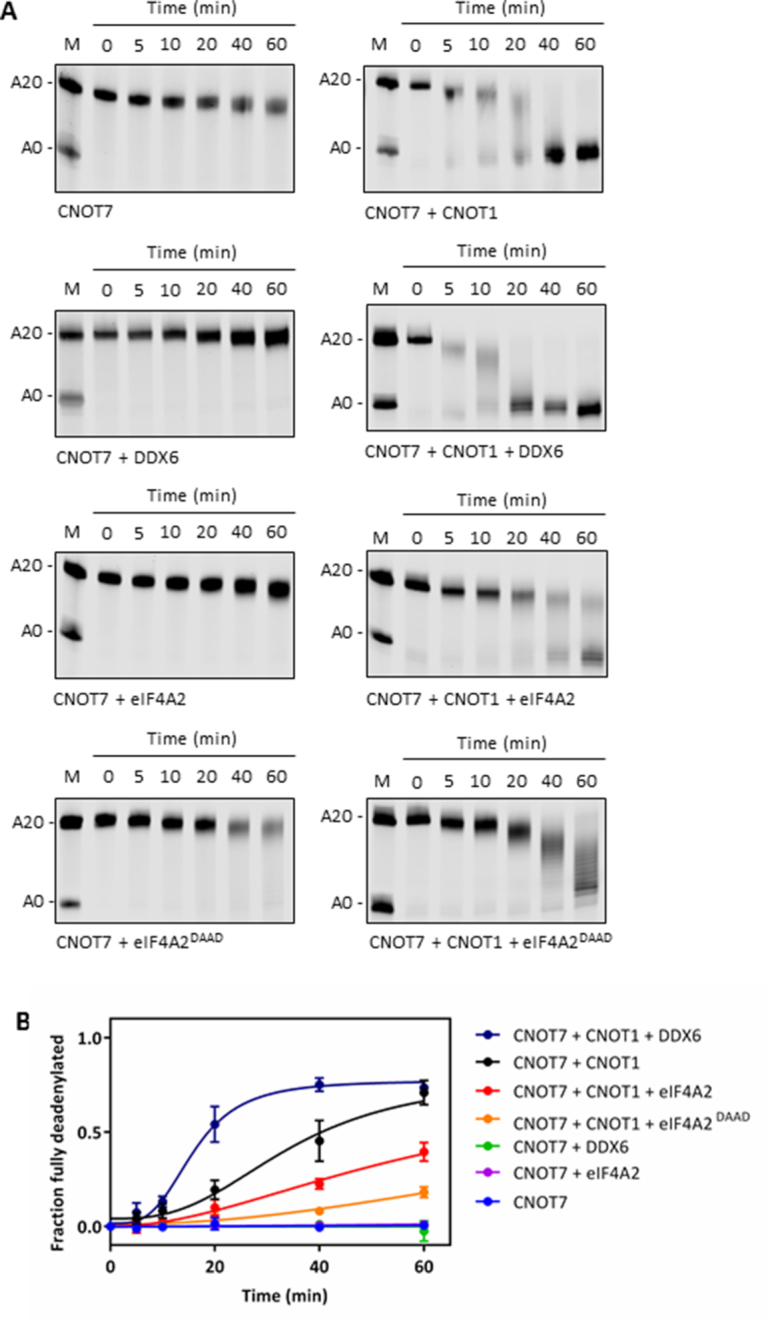
eIF4A2 inhibits CNOT7 deadenylation activity. (**A**) *In vitro* deadenylation assays were performed with recombinant proteins and 5′-Dy780-labelled RNA. Aliquots were taken after indicated time points and resolved on denaturing TBE–urea gels. M = substrate + deadenylated product marker. Representative gels are shown. (**B**) Bands were quantified and the fraction of fully deadenylated product over time plotted as mean ± sd, *n* = 3 biological repeats.

**Figure 7.**
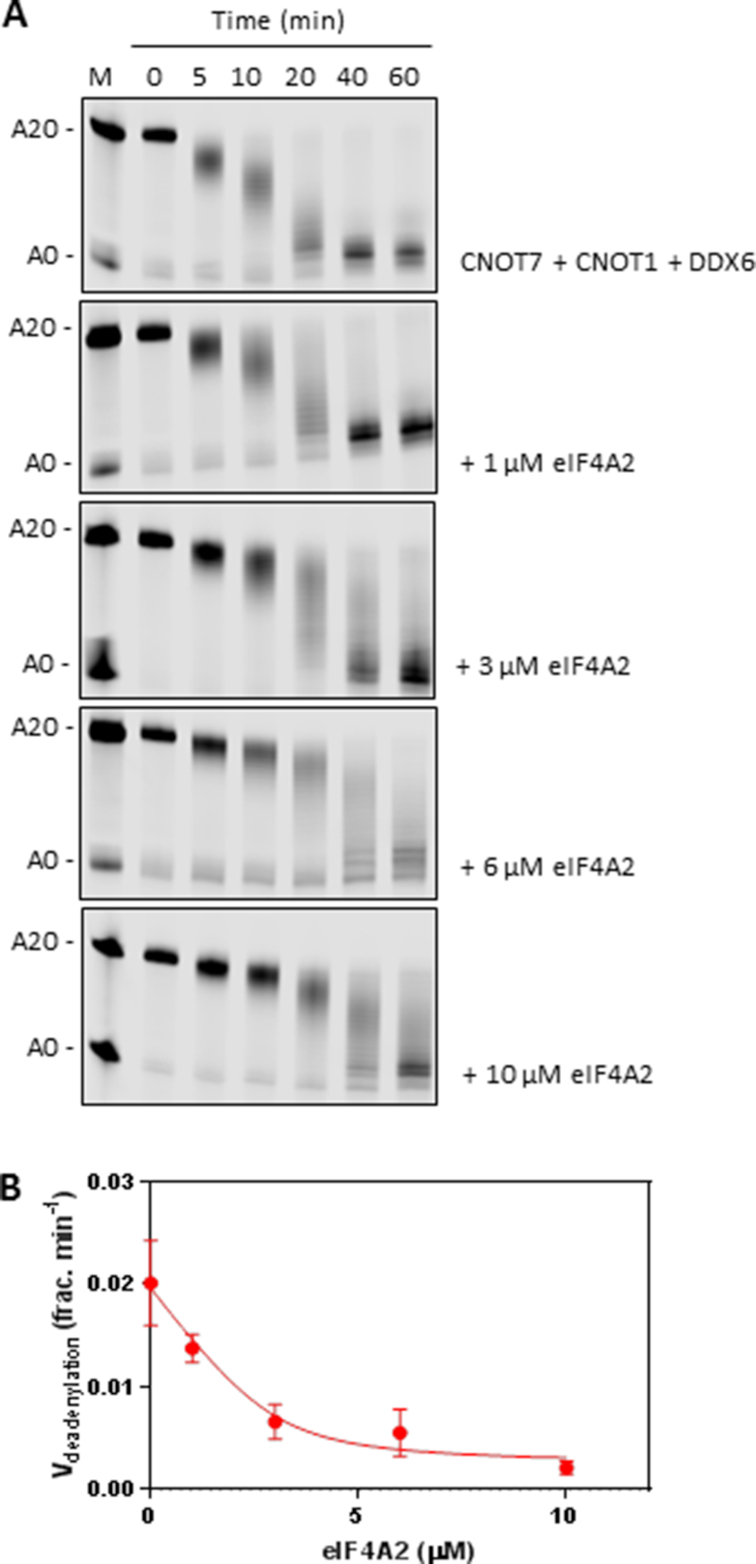
eIF4A2 and DDX6 compete to inhibit/promote deadenylation. (**A**) *In vitro* deadenylation assays were performed as in Figure 6A with increasing amounts of eIF4A2 as indicated. Representative gels are shown. (**B**) Bands were quantified and deadenylation speed was plotted as mean ± sd, *n* = 3 biological repeats.

To establish the role of eIF4A2 and DDX6 in the CCR4–NOT complex we performed *in vitro* deadenylation assays. CNOT7 alone was a very poor deadenylase (Figure 6A; 5,6,44,45) but CNOT7 activity was stimulated in the presence of the MA3 and MIF domains of CNOT1 (Figure 6A and B). DDX6 stimulated CNOT7 deadenylation activity via CNOT1, however, eIF4A2 had a strong inhibitory effect on CNOT7 (Figure 6A and B). eIF4A1 has a similar effect as eIF4A2 (Supplementary Figure S10A) and *in vitro* experiments using recombinant proteins eIF4A1 shows that it can interact with CNOT1 (Supplementary Figure S10B). However, we do not observe eIF4A1 interacting strongly with CNOT1 *in vivo* (Figure 2A) suggesting that in isolation these proteins can interact but that they are not interacting under physiological conditions. To further evaluate the ability of the helicases to interact specifically we conducted an immunoprecipitation experiment using recombinant proteins and found that a truncated version of eIF4G containing the MIF and MA3 domains (eIF4G-MIF-MA3) could interact with eIF4A1, eIF4A2 and DDX6, therefore, no specificity was observed *in vitro* (Supplementary Figure S10C). However, in the cell eIF4A1 is predominantly interacting with eIF4G and in this role it is involved in stimulating translational efficiency rather than the regulation of CNOT7 efficiency. To exclude the possibility that eIF4A2 is binding to the RNA substrate and preventing access to the poly(A) tail we repeated the deadenylation assay with an eIF4A2 mutant that cannot bind RNA (eIF4A2^DAAD^; Supplementary Figure S10D; 46). This mutant still repressed deadenylation, therefore it is the interaction of eIF4A2 with CNOT1 that is critical for inhibition of CNOT7 (Figure 6A and B). Because eIF4A2 and DDX6 compete for binding to CNOT1 this would predict that including both helicases in the deadenylation assay has a competitive effect. Indeed, when increasing amounts of eIF4A2 were added to a deadenylation assay containing CNOT1, CNOT7 and DDX6 the deadenylation rate decreased with increasing amounts of eIF4A2 (Figure 7A and B).

**Figure 8.**
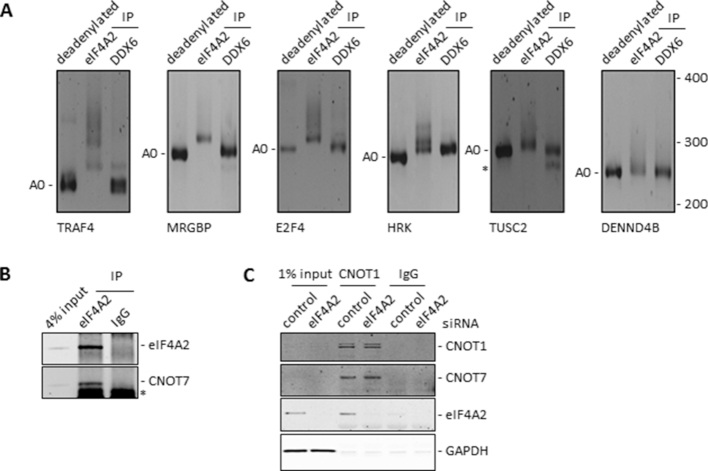
eIF4A2 bound mRNAs have a longer poly(A) tail than DDX6 bound mRNAs. (**A**) HEK293 RNA was immunoprecipitated with the indicated antibodies and analysed by poly(A) test (PAT) for six different targets. Deadenylated RNA was generated by incubating total RNA with RNaseH and oligo(dT). A0 indicates deadenylated PCR product. *Indicates a non-specific PCR product. (**B**) HEK293 cells were lysed and lysates were used for immunoprecipitation and analysed by western blotting. Representative blots are shown. *Indicates a non-specific reaction with the light chain. (**C**) HeLa cells were treated with siRNAs as indicated. Cells were lysed after 72 h and the resulting lysates were immunoprecipitated. Resulting complexes were eluted using CNOT1 peptide and analysed by western blotting. Representative blots are shown. GAPDH is a loading control.

These results implied that mRNAs bound to the CNOT1–eIF4A2 complex should have a longer poly(A) tail than mRNAs that are bound to the CNOT1–DDX6 complex. To test this hypothesis, we performed poly(A) tests (PAT) on eIF4A2 and DDX6 bound mRNAs (Figure 8A). Endogenous eIF4A2 and DDX6 immunoprecipitations identified several mRNAs that have the ability to bind both eIF4A2 and DDX6 (unpublished data Wilczynska *et al.*). Endogenous RNA immunoprecipitations were performed for eIF4A2 and DDX6 and the poly(A) tail length of associated mRNAs was determined by PAT assay. Target mRNAs for the PAT assay were selected based on high abundance and their ability to bind both eIF4A2 and DDX6 efficiently. eIF4A2 bound mRNAs had varying poly(A) tail lengths whilst DDX6 bound mRNAs had a very short or no poly(A) tail (Figure 8A, Supplementary Figure S11A). The variability in poly(A) tail length amongst the eIF4A2 bound mRNAs was a reflection of the average poly(A) tail length of total mRNA in the cell (Supplementary Figure S11B). mRNAs that bind exclusively to eIF4A2 had a varied poly(A) tail length, similar to the mRNAs that could bind both eIF4A2 and DDX6, showing again that eIF4A2 inhibited deadenylation (Supplementary Figure S12). To determine whether the lack of deadenylation activity of the eIF4A2–CNOT1–CNOT7 complex was caused by eIF4A2 interfering with the CNOT1–CNOT7 interaction we employed eIF4A2 immunoprecipitations which showed a clear interaction of eIF4A2 with CNOT7 (Figure 8B), as demonstrated before (15). We then used recombinant protein immunoprecipitations using different combinations of CNOT1, CNOT7 and eIF4A2 or DDX6 to analyse if these proteins affected each other's affinity for CNOT1 (Supplementary Figure S11C). These CNOT1 immunoprecipitations showed that eIF4A2 and DDX6 do not inhibit CNOT7 binding to CNOT1 and that CNOT7 does not affect the binding of eIF4A2 or DDX6 to CNOT1. Moreover, knockdown of eIF4A2 in HeLa cells followed by immunoprecipitation of CNOT1 showed that the presence or absence of eIF4A2 in the CCR4–NOT complex does not affect CNOT7 binding to CNOT1 (Figure 8C). The same was observed when eIF4A2 incorporation in the complex was increased following TAB182 knockdown (Figure 5D and E). Together, these data revealed that the repression of deadenylation activity of the eIF4A2–CNOT1–CNOT7 complex was not a consequence of diminished CNOT7 binding suggesting that eIF4A2 affects either CNOT7 deadenylation function or the ability of CNOT7 to access the poly(A) tail.

**Figure 9.**
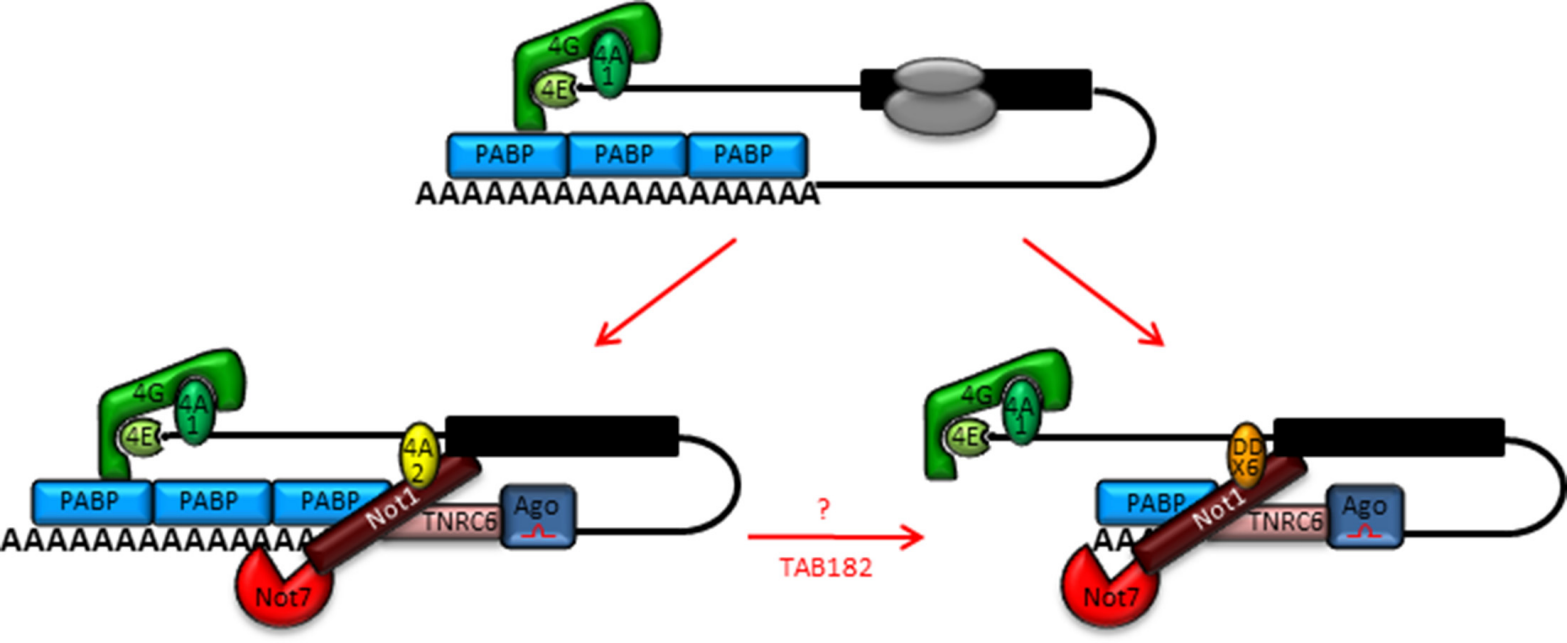
eIF4A2 inhibits CNOT7 deadenylation activity. An actively translated mRNA is bound by several initiation factors, including eIF4A1 (top). When the CCR4–NOT complex is recruited the mRNA is translationally repressed. If the CCR4–NOT complex includes eIF4A2 the poly(A) tail remains intact (bottom left). If the CCR4–NOT complex includes DDX6 instead the mRNA can be deadenylated (bottom right). TAB182 decreases eIF4A2 incorporation into the CCR4–NOT complex and therefore could affect the poly(A) tail length of the mRNA.

Together, these data show that eIF4A2 and DDX6 have distinct functions when interacting with the CCR4–NOT complex resulting in a different outcome for the targeted mRNA as described in the model shown in Figure 9. When the eIF4A2–CNOT1 complex is recruited to an mRNA it remains polyadenylated. In contrast, when the DDX6–CNOT1 complex gets recruited it results in deadenylation of the mRNA. The exact composition of the CCR4–NOT complex varies dependent on the circumstances. Each CCR4–NOT complex contains two deadenylases: CNOT7 or CNOT8 binds directly to the MIF domain of CNOT1 and CNOT6 or CNOT6L binds via CNOT7/8 resulting in four possible combinations of deadenylases in the complex (13). These deadenylases cooperate with PABP to determine deadenylation efficiency (5,6). Whilst CNOT7/8 removes the poly(A) tail which is not bound by PABP, CNOT6/6L is responsible for displacing PABP resulting in a cycling mechanism of poly(A) removal. Reduced PABP binding of miRNA targeted mRNAs renders them particularly vulnerable to CNOT7/8 deadenylation (5). Our data showed that eIF4A2 and DDX6 bind CNOT1 directly via the MA3 and MIF domains of CNOT1. Moreover, DDX6 in complex with CNOT1 stimulates CNOT7 deadenylation activity whilst eIF4A2 in complex with CNOT1 inhibits CNOT7 activity resulting in markedly different outcomes for the targeted mRNA (Figure 9). Our data suggest that of the mRNAs that can bind eIF4A2 and DDX6, TAB182 availability could determine which helicase will be incorporated into the CCR4–NOT complex. The fate of each mRNA is dependent on the choice of recruitment of eIF4A2 or DDX6 to the CCR4–NOT complex and the subsequent effect on the deadenylation of the mRNA.

## Supplementary Material

gkz509_Supplemental_Files

## Notes

Present address: Hedda A. Meijer, Division of Cell and Developmental Biology, School of Life Sciences, University of Dundee, Dundee DD1 5EH, UK.

Present address: Tobias Schmidt, Sarah L. Gillen, Ania Wilczynska and Martin Bushell, Cancer Research UK (CRUK), Beatson Institute, Glasgow G61 1BD, UK.

Present address: Claudia Langlais, Immunocore, Milton Park, Abingdon OX14 4RW, UK.
