# Supplementary material for: DEAD-box helicase eIF4A2 inhibits CNOT7 deadenylation activity
Source: Nucleic Acids Res. 2019 Jun 10;47(15):8224–38. doi: 10.1093/nar/gkz509 (PMC6736043; doi:10.1093/nar/gkz509)
Supplement: gkz509_Supplemental_Files [file gkz509_supplemental_files.zip › Meijer et al suppl.doc]

Supplementary materials for

**DEAD-box helicase eIF4A2 inhibits CNOT7 deadenylation activity**

Hedda A. Meijer, Tobias Schmidt, Sarah L. Gillen, Claudia Langlais, Rebekah Jukes-Jones, Cornelia H. de Moor, Kelvin Cain, Ania Wilczynska, Martin Bushell

**Fig. S1 Tethering of CNOT1 represses translation (A)** Schematic representation of the constructs used in the tethering assay. **(B)** HeLa cells were transfected with the plasmids from Fig. S1A 24 hours after plating. Cells were lysed in PLB after a further 48 hours and analysed by luciferase assay. Luciferase activity was plotted as average +/- sd, n=3 biological repeats. Significance was calculated using a student’s T-test (paired, 2-tailed); **p<0.01, n.s.= not significant. **(C)** Lysates from Fig. S1B were analysed by western blotting.

**Fig. S2 Knockdown of the helicases does not affect mRNA levels in the let-7 reporter and tethering assay. The relief of miRNA mediated repression following knockdown is not siRNA specific. (A)** Lysates from Fig. 1C were analysed by western blotting. **(B)** Cells were treated in parallel with Fig. 1C and analysed by qPCR, n=4 biological repeats. **(C)** Lysates from Fig. 1F were analysed by western blotting. **(D)** Cells were treated in parallel with Fig. 1F and analysed by qPCR, n=3 biological repeats. **(E)** HEK293 cells were treated and analysed as in Fig. 1C using alternative siRNAs, n=3 biological repeats. Significance was calculated using a student’s T-test (paired, 2-tailed); *p<0.05, **p<0.01. **(F)** Lysates from Fig. S2E were analysed by western blotting. **(G)** Cells were treated in parallel with Fig. S2E and analysed by qPCR, n=3 biological repeats. For western blotting representative blots are shown. GAPDH is a loading control. *Indicates a non-specific band.

**Fig. S3 eIF4A2 and DDX6 both interact directly with CNOT1. (A)** Western blotting analysis of recombinant eIF4A1 and eIF4A2. Quantification of the signal is indicated underneath each band. Representative blots are shown. **(B/C)** HeLa cells were transfected with siRNAs as indicated and analysed using PLA with the indicated antibodies after 48 hours and imaged using a confocal microscope. PLA dots indicate CNOT1-eIF4A2 **(B)** or CNOT1-DDX6 **(C)** interaction. Representative images are shown. **(D)** Western blotting analysis of cells treated in parallel with cells from Fig. S3B-C. Representative blots are shown. GAPDH is a loading control.

**Fig. S4 The CNOT1 MA3 and MIF domains are required for maximum helicase binding. (A)** Western blot analysis of the lysates from Fig. 3B. Representative blots are shown. GAPDH is a loading control. **(B)** Cells were treated in parallel with Fig. 3B and analysed by qPCR, n=4 biological repeats. **(C)** Immunoprecipitations with recombinant proteins performed in parallel with Fig. 2C using CNOT1-MIF. A representative gel is shown.

**Fig. S5 Mutation of the binding sites prevents binding, knockdowns are efficient and RNA levels are not affected. (A)** HeLa cells were transfected with the constructs used in Fig. 4A as well as HA-MA3-MIF (WT and mut4G23) and the resulting HA-tagged proteins were pulled down after 24 hours and analysed by western blotting. **(B)** Western blot analysis of the lysates from Fig. 4A. **(C)** Cells were treated in parallel with Fig. 4A and analysed by qPCR, n=3 biological repeats. **(D)** HeLa cells were transfected with the constructs used in Fig. 4B and resulting HA-tagged proteins were pulled down after 24 hours and analysed by western blotting. **(E)** Lysates from Fig. 4B were analysed by western blot. **(F)** Cells were treated in parallel with Fig. 4B and analysed by qPCR, n=3 biological repeats. GAPDH is a loading control.

**Fig.S6 eIF4A2 and DDX6 contribute equally to translational repression via the CNOT1 MIF domain. (A)** HeLa cells were transfected and analysed as in Fig. 1F with the constructs depicted in Fig. 3A (right hand panel) and analysed by luciferase assay, n=3 biological repeats. Significance was calculated using a student’s T-test (paired, 2-tailed); *p<0.05, **p<0.01. **(B)** Lysates from Fig. S6A were analysed by western blotting. Representative blots are shown. GAPDH is a loading control.

**Fig. S7 Preparation of samples for MS identification of components of the CNOT1-eIF4A2 complex. (A)** Cytoplasmic HeLa lysate was fractionated using a Sephacryl S-500 HR column connected to an AKTA purifier protein purification system. A280 was monitored. **(B)** Fractions from Fig. S7A were analysed by western blotting. Every third fraction of all 67 fractions of the gel filtration column was loaded on the gel. The red box indicates the fractions containing CNOT1. **(C)** Unfractionated HeLa lysate and gel filtration fractions 30-32 were analysed by western blotting. Unfractionated lysate panels and fractions panels are part of the same exposure of the same blot. Quantification of the relative band intensity is indicated underneath the panels. **(D)** Recombinant eIF4A1 and eIF4A2 proteins and HeLa lysate were analysed by western blotting. Quantification of the signal is indicated underneath each band. **(E)** Fractions 30-32 were subjected to immunoprecipitation in the presence of RNaseA. Protein complexes were eluted using CNOT1 peptide and analysed by western blotting to check for the presence of the CNOT1-eIF4A2 complex. **(F)** Fractions 30-32 and cleared HeLa lysate as used for loading of the S500 column were treated with RNaseA for the duration of the immunoprecipitationin Fig. S7E. **(G)** HeLa cells were transfected with siRNAs as indicated. After 72 hours the cells were lysed and subjected to immunoprecipitation. Protein complexes were eluted from the beads using a CNOT1 peptide and analysed using western blotting. **(H)** Fractions 30-32 were subjected to immunoprecipitation in the presence of RNaseA. Immunoprecipitates were separated using SDS-PAGE and detected using ProtoBlue staining. The resulting gels were sliced and analysed by MS (see Table S4). **(I)** Fractions 30-32 were treated as in Fig. S7H. Protein complexes were eluted using CNOT1 peptide. For MS analysis see Table S5. For consolidated results after combination of both sets of MS data see Table 1. For western blotting representative blots are shown.

**Fig. S8 Validation of MS results. (A)** Cytoplasmic HeLa lysate was immunoprecipitated using the antibodies indicated in the presence of RNAseA and analysed by western blotting. Pat1b is a negative control. **(B)** Cytoplasmic HeLa lysate was fractionated as in Fig. S7A and fractions analysed by western blotting. For western blotting representative blots are shown.

**Fig. S9 TAB182 binds to eIF4A2 but not eIF4A1. (A)** Cytoplasmic HeLa lysates were immunoprecipitated in the presence of RNAseA and analysed by western blotting. **(B)** Recombinant eIF4A1 and eIF4A2 proteins and HeLa lysate were analysed by western blotting. eIF4A1 and eIF4A2 panels are part of the same exposure of the same blot. Quantification of the signal is indicated underneath each band. Representative blots are shown. **(C)** Lysates from Fig. 5C were analysed by western blotting. GAPDH is a loading control. *Indicates a non-specific band. **(D)** HEK293 cells were treated in parallel with Fig. 5C and analysed by qPCR, n=3 biological repeats. For western blotting representative blots are shown.

**Fig. S10 eIF4A1 and eIF4A2 RNA binding mutant inhibit deadenylation. (A)** *In vitro* deadenylation assays using eIF4A1 were performed in parallel with Fig 6A. **(B/C)** Immunoprecipitations with recombinant proteins performed as in Fig. 2C. **(D)** Electrophoretic mobility shift assays of eIF4A2^wt^ and eIF4A2^DAAD^ RNA binding mutant in the presence of labelled ssRNA (CAA)_6_CA-C_20_-A_20_ RNA.

**Fig. S11 Some mRNAs bound to eIF4A2 and DDX6 have a very short poly(A) tail in HeLa cells. eIF4A2, DDX6 and CNOT7 do not affect each other’s interaction with CNOT1. (A)** A fraction of the immunoprecipitate from Fig. 8A was analysed by western blotting. Representative blots are shown. **(B)** Deadenylated and untreated poly(A)+ RNA were analysed by PAT as in Fig. 8A. *Indicates a non-specific PCR product. **(C)** Recombinant eIF4A2, DDX6 and CNOT7 proteins were incubated with recombinant CNOT1-MA3-MIF in the presence of ssRNA (CAA)_6_CA-C_20_-A_20_ and AMPPNP and subsequently pulled down using the HIS-tag of CNOT1 and analysed by denaturing SDS-PAGE and Coomassie-staining. A representative gel is shown.

**Fig S12 Poly(A) tail length of an mRNA is dependent on which helicase it binds. (A/B)** mRNAs that can bind to eIF4A2 only were analysed by PAT assay as in Fig. 7A. Deadenylated and immunoprecipitated mRNA (A) or deadenylated and untreated poly(A)+ RNA (B) were analysed. **(C/D)** mRNAs that can bind to DDX6 only were analysed by PAT assay. Deadenylated and immunoprecipitated mRNA (C) or deadenylated and untreated poly(A)+ RNA (D) were analysed. A0 indicates deadenylated PCR product. *Indicates a non-specific PCR product.
